# Ruxolitinib inhibits poly(I:C) and type 2 cytokines‐induced CCL5 production in bronchial epithelial cells: A potential therapeutic agent for severe eosinophilic asthma

**DOI:** 10.1002/iid3.397

**Published:** 2021-02-03

**Authors:** Mitsuru Sada, Masato Watanabe, Toshiya Inui, Keitaro Nakamoto, Aya Hirata, Masuo Nakamura, Kojiro Honda, Takeshi Saraya, Daisuke Kurai, Hirokazu Kimura, Haruyuki Ishii, Hajime Takizawa

**Affiliations:** ^1^ Department of Respiratory Medicine Kyorin University School of Medicine Tokyo Japan; ^2^ Division of Infectious Diseases, Department of General Medicine, School of Medicine Kyorin University Tokyo Japan; ^3^ Department of Health Science Graduate School of Health Science, Gunma Paz University Gunma Japan

**Keywords:** CCL5, eosinophilic asthma, ruxolitinib, bronchial epithelium, IL‐13

## Abstract

**Rationale:**

Severe eosinophilic asthma is characterized by airway eosinophilia and corticosteroid‐resistance, commonly overlapping with type 2 inflammation. It has been reported that chemokine (C‐C motif) ligand 5 (CCL5) is involved in the exacerbation of asthma by RNA virus infections. Indeed, treatment with a virus‐associated ligand and a T helper type 2 cell (Th2) cytokine can synergistically stimulate CCL5 production in bronchial epithelial cells. We aimed to evaluate the mechanisms underlying CCL5 production in this in vitro model and to assess the potential of Janus kinase 1 (JAK1) as a novel therapeutic target via the use of ruxolitinib.

**Methods:**

We stimulated primary normal human bronchial epithelial (NHBE) cells and BEAS‐2B cells with poly(I:C) along with interleukin‐13 (IL‐13) or IL‐4, and assessed CCL5 production. We also evaluated the signals involved in virus‐ and Th2‐cytokine‐induced CCL5 production and explored a therapeutic agent that attenuates the CCL5 production.

**Results:**

Poly(I:C) stimulated NHBE and BEAS‐2B cells to produce CCL5. Poly(I:C) and IL‐13 increased CCL5 production. Poly(I:C)‐induced CCL5 production occurred via the TLR3–IRF3 and IFNAR/JAK1–phosphoinositide 3‐kinase (PI3K) pathways, but not the IFNAR/JAK1–STATs pathway. In addition, IL‐13 did not augment poly(I:C)‐induced CCL5 production via the canonical IL‐13R/IL‐4R/JAK1–STAT6 pathway but likely via subsequent TLR3‐IRF3‐IFNAR/JAK1‐PI3K pathways. JAK1 was identified to be a potential therapeutic target for severe eosinophilic asthma. The JAK1/2 inhibitor, ruxolitinib, was demonstrated to more effectively decrease CCL5 production in BEAS‐2B cells than fluticasone propionate.

**Conclusion:**

We have demonstrated that JAK1 is a possible therapeutic target for severe corticosteroid‐resistant asthma with airway eosinophilia and persistent Th2‐type inflammation, and that ruxolitinib has potential as an alternative pharmacotherapy.

## Graphical abstract

First, we focused on whether treatment with a virus‐associated ligand and type‐2 cytokine can synergistically stimulate CCL5 production in bronchial epithelial cells, in which we confirmed the synergistic effect. Second, we evaluated the mechanisms underlying the synergistical CCL5 production and found that the JAK1 is a novel therapeutic target for severe eosinophilic asthma. Finally, we have demonstrated that ruxolitinib is a potential therapeutic agent for severe corticosteroid‐resistant asthma with airway eosinophilia and persistent Th2‐type inflammation.

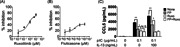

## 1 INTRODUCTION

Bronchial asthma is characterized by chronic airway inflammation, leading to expiratory airflow limitation and presentation of respiratory symptoms (e.g., dyspnea and wheezing).^1^ A small proportion of patients with asthma (5%–10%) can be classified as having severe asthma, in which symptoms remain uncontrolled despite the administration of high‐dose inhaled corticosteroids (i.e., fluticasone propionate [FP]) in combination with a second long‐term controller medication.^2^ These patients represent a substantial economic burden owing to their symptoms, disease exacerbation, and medication‐induced side effects, accounting for greater than 60% of the medical costs associated with asthma.^3^ While eosinophilic asthma with persistent type 2 inflammation constitutes the most common phenotype of severe asthma, there is limited knowledge regarding the pathophysiology of refractory eosinophilic asthma.

Eosinophilic airway infiltration plays an important role in the pathogenesis of asthma. Various Th2 cytokines and chemokines recruit intravascular eosinophils to airway.^4^ Airway eosinophilia is associated with recurring bronchial hyperresponsiveness and airflow limitations, which respectively account for the pathogenesis and severity of asthma.^5^ Airway eosinophil counts are higher in asthmatics than in healthy subjects, and are further elevated following viral infection.^6^ Indeed, many reports have suggested that respiratory viral infections are associated with the onset and/or exacerbation of asthma. This is termed virus‐induced asthma, and infection with a respiratory virus may be associated with greater than 80% of asthma cases.^7^ The most common causes of virus‐induced asthma are human rhinovirus, respiratory syncytial virus (RSV), and enteroviruses (EV), all of which are RNA viruses.^7^, ^8^, ^9^, ^10^ In general, innate immunity plays an important role in the primary immune response against viruses.^11^, ^12^ Such immunity to RNA viruses reportedly involves various Toll‐like receptors (TLR), such as TLR3 and TLR7/8,^13^ NOD‐like receptors (NLRs), and retinoic acid inducible gene‐I ‐like receptors (RLRs), all of which are activated by the viral RNA.^11^ In infected cells, these RNA viruses synthesize double‐strand (dsRNA) during replication. These dsRNAs are recognized by TLR3, NLR, and RLR, although one study suggests that TLR3 is the main receptor of dsRNA in airway epithelial cells using poly(I:C) which is a viral dsRNA analog.^14^ In contrast, single‐strand RNA is recognized by TLR7/8. However, it has been reported that TLR7 suppresses T helper type 2 cell (Th2) responses and inhibits allergic airway diseases.^15^ Thus, immune responses involving TLR3are associated with the pathophysiology of asthma, although the precise mechanisms for this are not yet fully understood.

Th2 cytokines, including interleukin (IL)‐4 and IL‐13, are closely related to various allergic diseases including asthma.^16^ Numerous reports have shown that IL‐13 causes exacerbation of asthma.^17^, ^18^, ^19^ This cytokine is produced by tissue Th2 lymphocytes and innate lymphoid 2 cells, which act on the allergic immune cells (e.g., eosinophils and mast cells), inducing their migration from the vessel.^20^, ^21^ Moreover, IL‐13 can induce a chemokine, Chemokine (C‐C motif) ligand 5 (CCL5), from various cells including airway epithelial cells.^22^, ^23^ CCL5 attracts T cells, its expression is regulated by activated T cells, and it has strong chemotactic activity for eosinophils.^24^ Furthermore, Th2 cytokines have synergistic effects on CCL5 production in air way cells.^25^ Thus IL‐13 and CCL5 may be associated with asthma exacerbation, although this relationship remains unclear.^16^, ^26^, ^27^

Furthermore, ruxolitinib, a Janus kinase 1 (JAK1) and JAK2 subtype inhibitor, is used as a molecular targeted agent for the treatment of osteofibrosis.^28^, ^29^ It has been reported that the JAK1 pathway is involved in the production of inflammatory cytokines and interferon via the TLRs.^30^, ^31^ In addition, IL‐13 can activate JAK1.^32^ TLR3 and IL‐13 may induce the phosphorylation of JAK1, resulting in allergic reactions. Thus, ruxolitinib may regulate the allergic reaction induced by TLRs and IL‐13 in the airway cells, leading to asthma remission.

Thus, the objective of this study was to clarify whether ruxolitinib disrupts the relationship between the innate immunity that is induced by poly(I:C), the allergic cytokine IL‐13, and the chemokine CCL5 in airway epithelial cells (BEAS‐2B cells). We found that ruxolitinib was a better inhibitor than FP for decreasing the synergistic production of CCL5 by poly(I:C) and IL‐13 in vitro.

## 2 MATERIALS AND METHODS

### 2.1 Reagents

It is well known that poly(I:C) stimulates innate immunity such as TLR3.^33^ Thus, it was used for surrogate viral RNA such as rhinovirus.^13^ We also used CpG oligonucleotides (CpG–ODN), TLR9 ligand replacement of viral DNA. Both nucleic acid compounds were purchased from Novus Biologicals. IL‐13 and IL‐4 were purchased from PeproTech. IL‐33 was purchased from Wako Pure Chemical. IL‐37 and CC16 (Clara cell secretory protein; a marker of bronchial lung epithelial cells) were purchased from ProSpec and R&D Systems, respectively. BAY 11‐7082 (an inhibitor of nuclear factor kappa light chain (NF‐κB) enhancer of activated B cells was purchased from InvivoGen. Ruxolitinib, stattic (an inhibitor of signal transducer and activator of transcription 3^34^), and LY294002 (a phosphatidylinositol kinase‐3 inhibitor) were purchased from Cayman Chemical. The chemotherapy agent, fludarabine, was purchased from Wako Pure Chemical. The corticosteroid, FP, was purchased from Sigma. A type I interferon (IFNs) neutralizing antibody mixture was purchased from PBL Assay Science. Small interfering RNAs (siRNAs) for TLR3, interferon regulatory factor (IRF) 3, RelA (a NF‐κB subunit), JAK1, STAT6, extracellular signal‐regulated kinase (ERK) 1, ERK2, Stealth RNAi siRNA Negative Control Med GC Duplex #2, and Lipofectamine RNA iMAX Reagent were purchased from Invitrogen.

### 2.2 Cell culture

BEAS‐2B cells, which are virus‐transformed human bronchial epithelial cells, and primary normal human bronchial epithelial (NHBE) cells were respectively purchased from ATCC and Lonza and cultured according to the suppliers' recommendations. Briefly, BEAS‐2B cells (2.0 × 10^5^ cells/well) and NHBE (1.0 × 10^4^ cells/well) cells were cultured with serum‐free bronchial epithelial growth media (Lonza) to a confluence of 80%–100%, which usually took 3 days. Cells were afterwards stimulated with cytokines and/or TLR ligands for 24 h in a 24‐well culture plate unless otherwise specified. For inhibitory assays, BEAS‐2B cells were co‐incubated with inhibitors for 2 h before cytokine stimulation. To evaluate the activity of inhibitors, percent (%) inhibition at each concentration of the inhibitor was calculated using the following equation:

$$\%\text{inhibition}=\left(1-A/B\right)\times 100,$$

where A and B were culture‐media CCL5 concentrations of BEAS‐2B cells grown with poly(I:C) after pre‐incubation treatment with an inhibitor and a vehicle, respectively. We then performed a curve fitting analysis using the following equation:

$$Y=\text{Bottom}+X\times \left(\text{Top}-\text{Bottom}\right)/\left(EC_{50}+X\right)$$

and calculated the maximal inhibitory effect of inhibitors.

### 2.3 Transfection of small interfering RNAs

BEAS‐2B cells, grown to 60%−80% confluence, were transfected with siRNAs or negative controls using the transfection reagent lipofectamine RNA iMAX for 2−3 days. This was done according to the manufacturer's instructions and was followed by cytokine stimulation. The knockdown efficiency was assessed by reverse transcription‐quantitative polymerase chain reaction.

### 2.4 ELISA

CCL5 was measured using an ELISA kit (Duoset, R&D Systems), following the manufacturer's instructions.

### 2.5 RNA isolation and real‐time polymerase chain reaction

Total RNA was isolated and purified by using a QIA shredder (QIAGEN) and RNeasy Mini kit (QIAGEN). Complementary DNA (cDNA) was synthesized using the following protocol. One microgram (μg) of total RNA, a random primer (Takara Bio), dNTP mix (Invitrogen), and distilled water were mixed and heated to 65°C for 5 min using the Gene Atlas 485 (ASTEC). SuperScript III RT (Invitrogen), 5X First‐Strand Buffer (Invitrogen), 0.1 M DTT (dithiothreitol; Invitrogen), and RNaseOUT (Invitrogen) were then added to the mixture, which was allowed to incubate at 55°C for 60 min and again at 75°C for 15 min using Gene Atlas 485. cDNA, QuantiTect SYBR Green PCR (QIAGEN), a specific primer, and distilled water were mixed and incubated as follows: 45 cycles at 95°C for 15 s, 55°C for 15 s, and 75°C for 20 s. The $∆∆C_{t}$ relative value method, using glyceraldehyde 3‐phosphate dehydrogenase (GAPDH) as the housekeeping gene, was utilized to calculate gene expression, after which the threshold cycle numbers were obtained using Stratagene Mx3000p (Agilent Technologies). The sequences of the specific primers were: CCL5 forward: 5ʹ‐TGA CCA GGA AGG AAG TCA GC‐3ʹ, reverse: 5ʹ‐AGC CGA TTT TTC ATG TTT GC‐3ʹ, GAPDH forward: 5ʹ‐TGA ACG GGA AGC TCA CTG G‐3ʹ, reverse: 5ʹ‐TCC ACC ACC CTG TTG CTG TA‐3ʹ, TLR3 forward: 5ʹ‐CTC AGA AGA TTA CCA GCC GCC‐3’, reverse: 5’‐CCA TTA TGA GAC AGA TCT AAT G‐3’.

### 2.6 The tetrazolium salt assay

The cytotoxic effect of ruxolitinib, LY294002, and FP over 24 h was evaluated using a tetrazolium salt assay, which involved 3‐(4,5‐dimethyl‐2‐thiazolyl)‐2,5‐diphenyltetrazolium bromide (MTT) (Wako Pure Chemical). This was dissolved in BEBM at a concentration of 0.5 mg/ml and incubated with the cells for 2 h. After the supernatant had been removed, dimethyl sulfoxide was added and the cells were placed on a shaking platform for 30 min. Absorbance at 570 nm was then measured for duplicate 100 μl samples using an iMark Microplate Reader.

### 2.7 Statistical analysis

All data are shown as mean ± standard error. Three separate replicates of each experiment were performed to confirm reproducibility. When comparing two or more groups, we used the student *t* test or one‐way analysis of variance with post hoc Holm–Sidak's multiple tests for comparing selected pairwise measurements. *p* Values of less than .05 were considered statistically significant. All experiments were repeated at least twice, with similar results. For statistical analysis, GraphPad Prism ver. 7.00 was used.

## 3 RESULTS

### 3.1 Poly(I:C) potentiates CCL5 production in human bronchial epithelial cells

**Figure 1:**
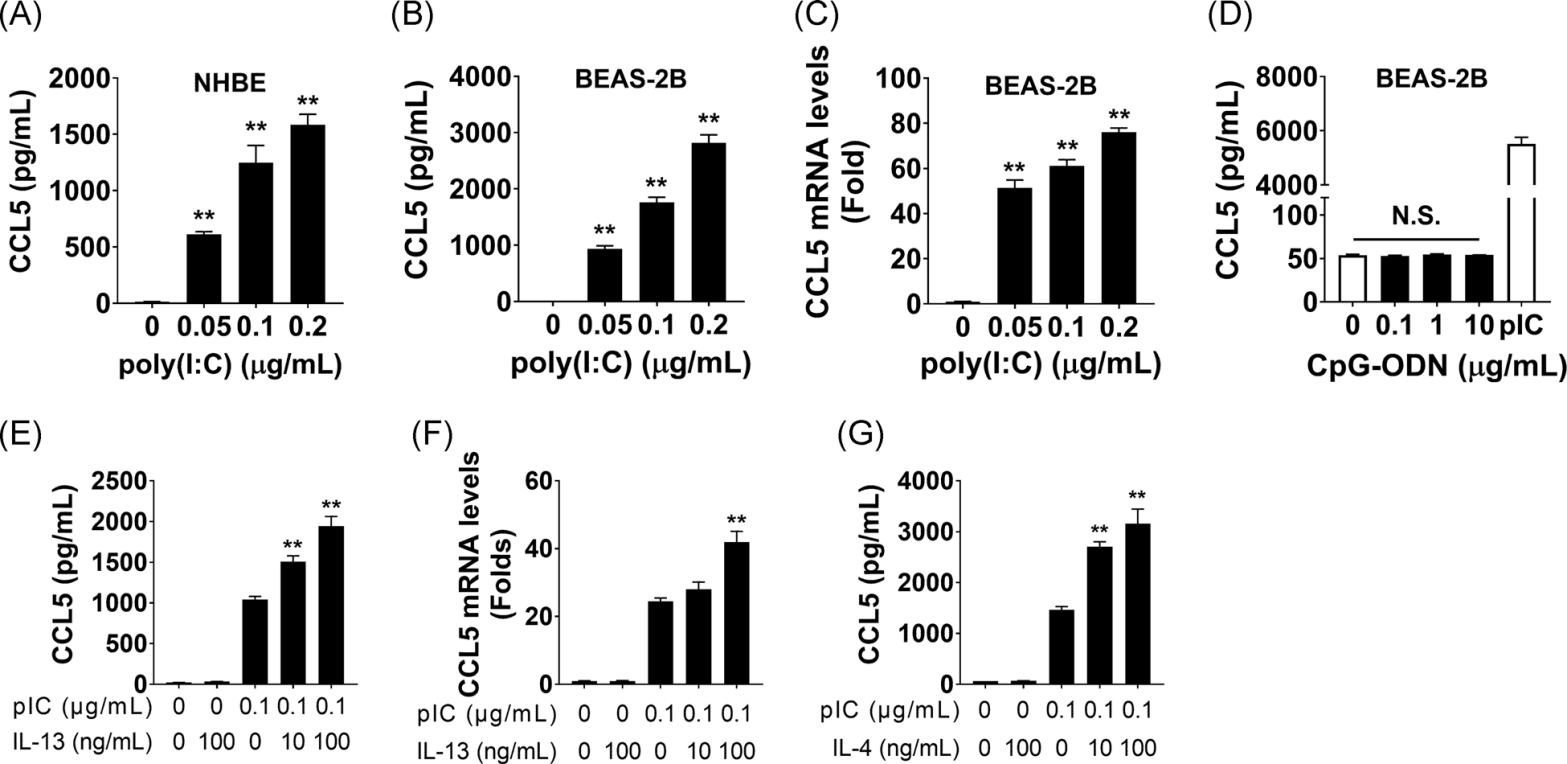
Poly(I:C) stimulates chemokine (C‐C motif) ligand 5 (CCL5) production in bronchial epithelial cells. (A) NHBE cells were stimulated with poly(I:C) for 24 h, and CCL5 levels were measured in the culture supernatant. (B–D) BEAS‐2B cells were stimulated with poly(I:C) or CpG–ODN as indicated for 24 h (B, D) or 12 h (C), and the CCL5 concentration in the culture supernatant (B, D) and CCL5 messenger RNA (mRNA) expression (C) were evaluated. (E–G) BEAS‐2B cells were stimulated with poly(I:C), interleukin‐13 (IL‐13), and IL‐4, as indicated, for 24 h (E, G) or 12 h (F), and the CCL5 concentration in the culture supernatant (E, G) and CCL5 mRNA expression (F) were evaluated. *p < .05, **p < .01, as compared to medium alone, using one‐way analysis of variance (ANOVA) with post hoc Holm–Sidak's multiple tests to conduct selected pairwise comparisons. NHBE, normal human bronchial epithelial; ODN, oligonucleotide; pIC, poly(I:C)

Initially, we examined whether bronchial epithelial cells produced CCL5 when stimulated with poly(I:C), a ligand for TLR3. We found that poly(I:C) enhanced CCL5 production in primary bronchial epithelial cells (Figure 1A). In BEAS‐2B cells, poly(I:C) stimulated both CCL5 protein release (Figure 1B) and messenger RNA (mRNA) expression (Figure 1C). For better reproducibility we used BEAS‐2B cells instead of primary bronchial cells for all subsequent culture experiments. We also stimulated BEAS‐2B cells with CpG–ODN, another viral ligand for TLR9, but this treatment did not augment cellular CCL5 production (Figure 1D). These results demonstrated that dsRNA enhanced CCL5 production in these lines of bronchial epithelial cells.

### 3.2 Poly(I:C)‐induced CCL5 production is further enhanced by the presence of Th2 cytokines

**Figure 2:**
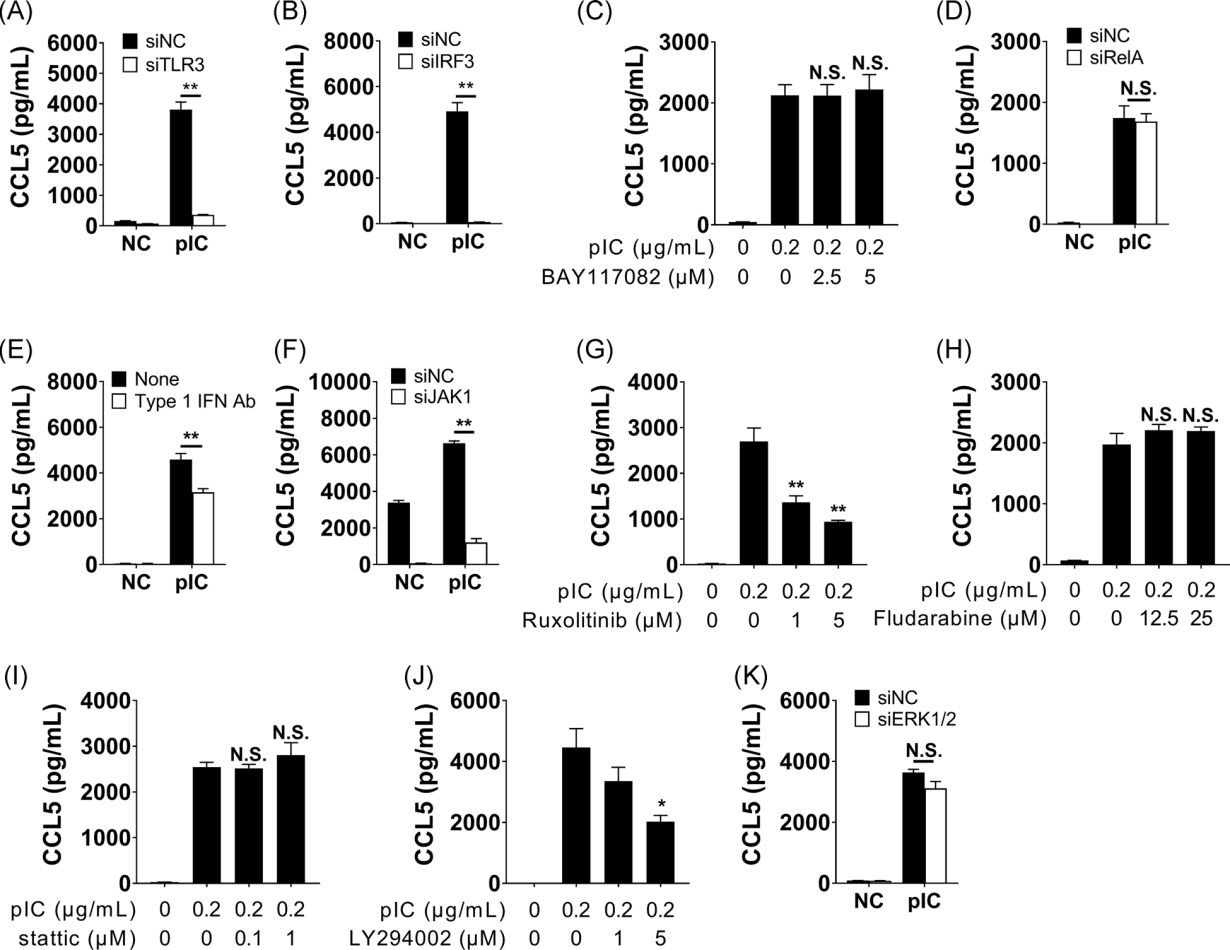
Signal transduction mechanisms in poly(I:C)‐induced CCL5 production in BEAS‐2B cells. (A–D) Of the TLR3‐related signals, si‐TLR3 (A) and si‐IRF3 (B), but neither NF‐κb inhibitor BAY117082 (C) nor si‐RelA (D) inhibited poly(I:C)‐induced CCL5 production. (E–I) In type I interferon (IFN)‐related signals, (E) neutralizing anti‐type I IFN antibody mixture, (F) si‐Janus kinase 1 (JAK1) (F), and JAK1/2 inhibitor ruxolitinib (G), but neither STAT1 inhibitor fludarabine (H) nor STAT3 inhibitor Stattic (I) attenuated poly(I:C)‐induced CCL5 production. (J, K) In alternative signals, phosphoinositide 3‐kinase (PI3K) inhibitor LY294002 (J) but not si‐Erk1/2 (K) reduced poly(I:C)‐induced CCL5 production. For all experiments, BEAS‐2B cells were transfected with small interfering RNAs (siRNAs) for 2 days (A–B, D, F, and K) or pre‐incubated with inhibitors for 2 h (C, E, G–I). Afterwards, these were stimulated with poly(I:C) (0.1 μg/ml) for 24 h, followed by measurement of CCL5 concentrations in the culture supernatant (A–I). *p < .05, **p < .01, as compared to medium alone. We used student *t* tests (A, B, D, F, and K) or one‐way ANOVA with post hoc Holm–Sidak's multiple tests to conduct selected pairwise comparisons of treatments (C, E, G‐I). ANOVA, analysis of variance; CCL5, chemokine (C‐C motif) ligand 5; NC, negative control; NF‐κb, nuclea factor κB; pIC, poly(I:C)

Next, we examined whether the presence of Th2‐type cytokines further stimulated poly(I:C)‐induced CCL5 production in bronchial epithelial cells. IL‐13 enhanced poly(I:C)‐induced CCL5 production (Figure 1E) and mRNA expression (Figure 1F) in BEAS‐2B cells. IL‐4 also augmented poly(I:C)‐induced CCL5 production (Figure 1G), even though neither IL‐13 nor IL‐4 alone stimulated CCL5 production in BEAS‐2B cells (Figures 1E,G). IL‐33, IL‐37, and CC16 in combination with poly(I:C) all failed to stimulate CCL5 production (Figure S1A–C). We also examined whether IL‐13 enhanced poly(I:C)‐induced CXCL8 production, but it did not (Figure 2). Together, these data show that poly(I:C) plus IL‐13 or IL‐4 synergistically induced CCL5 production in bronchial epithelial cells.

### 3.3 Signal transduction mechanisms in BEAS‐2B cells stimulated with poly(I:C) and IL‐13

**Figure 3:**
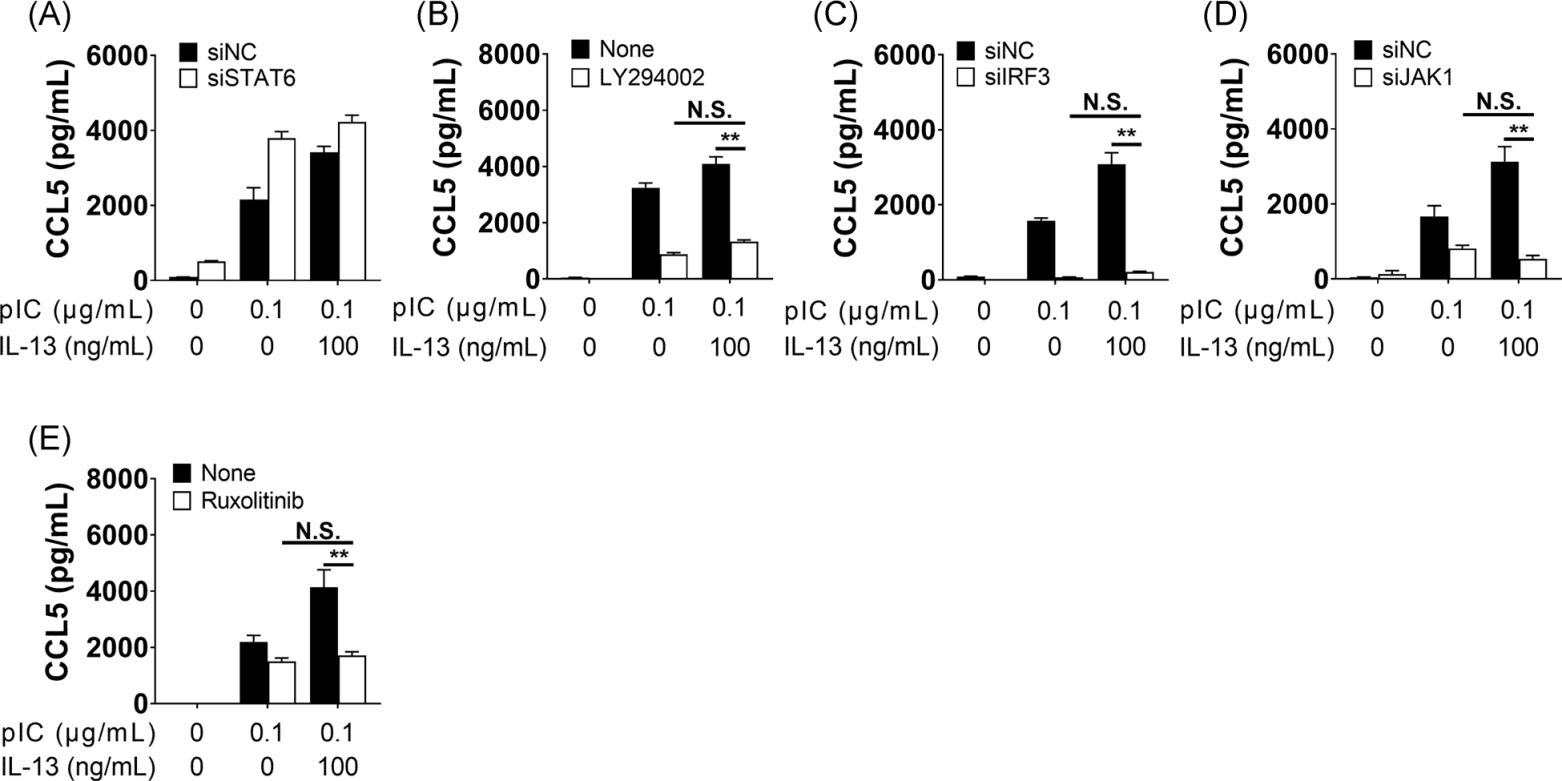
Signal transduction mechanisms in poly(I:C) and IL‐13‐induced CCL5 production in BEAS‐2B cells. (A–E) Poly(I:C) and IL‐13‐induced CCL5 production was not reduced with si‐STAT6 (A) but was inhibited with the PI3K inhibitor, LY294002 (5 µM, B). (C–E) si‐IRF3 (C), and si‐JAK1 (D). The JAK1/2 inhibitor, ruxolitinib (10 µM, E), also reduced poly(I:C) and IL‐13‐induced CCL5 production. BEAS‐2B cells were pre‐incubated with siRNA for 2 days (A, C, D) or with inhibitors for 2 h (B, E), followed by stimulation with poly(I:C) (0.1 μg/ml) for 24 h. *p < .05, **p < .01, as compared to medium alone. We used student *t* tests (A, C, D) or one‐way ANOVA with post hoc Holm–Sidak's multiple tests to conduct selected pairwise comparisons of treatments (B, E). ANOVA, analysis of variance; CCL5, chemokine (C‐C motif) ligand 5; IL, interleukin; JAK1, Janus kinase 1; NC, negative control; PI3K, phosphoinositide 3‐kinase; pIC, poly(I:C); siRNA, small interfering RNA

Using siRNA techniques and inhibitors, we investigated the signal transduction mechanisms that may be involved in CCL5 production of BEAS‐2B cells after stimulation with poly(I:C) and IL‐13. Before measuring CCL5 production, we conducted an MTT assay using inhibitors. There was no significant difference between the effect of poly(I:C) alone and that of poly(I:C) combined with 10 μM of ruxolitinib. On the other hand, there was a significant difference between poly(I:C) alone and poly(I:C) combined with 5 μM of LY294002, but the difference in OD570 was less than 10%. Therefore, we regard that combinations of these materials did not also affect cell viability (data not shown). We first examined molecular signals for CCL5 production as induced by poly(I:C) alone. TLR3‐ and IRF3‐knockdown strongly inhibited poly(I:C)‐induced CCL5 production (Figure 2A,B), whereas Rel A‐knockdown and NF‐κB inhibitor (BAY11‐7082) did not affect CCL5 production (Figure 2C,D). The neutralizing antibody against type I IFNs mixture inhibited poly(I:C)‐induced CCL5 production (Figure 2E). Next, we assessed pathways that were associated with the interferon receptor. We found that si‐JAK1 and the JAK1 inhibitor, ruxolitinib, attenuated poly(I:C)‐induced CCL5 production (Figure 2F,G). However, inhibitors for STAT1 and STAT3 did not affect poly(I:C)‐induced CCL5 production (Figure 2H,I). These findings suggest that canonical type I interferon receptor (IFNAR)/JAK–STAT‐associated pathways were not involved in the activation of poly(I:C)‐induced CCL5 production. We subsequently assessed alternative pathways, including phosphoinositide 3‐kinase (PI3K) and Erk1/2, and we found that PI3K, but not Erk1/2, is involved in poly(I:C)‐induced CCL5 production (Figure 2J,K). Therefore, the TLR3–IRF3–IFNAR/JAK1–PI3K cascade played an important role in poly(I:C)‐induced production of CCL5 in bronchial epithelial cells. ext, we evaluated the role of IL‐13 receptor‐associated signals in cells treated with both IL‐13 and poly(I:C). The canonical IL‐13 receptor signal activated the IL‐4Rα/IL‐13Rα1/JAK1–STAT6 pathway. We therefore performed STAT6 knockdown using siRNA techniques, reducing STAT6 mRNA levels to 12.7% (data not shown). STAT6‐knockdown failed to inhibit the synergistic effect observed from poly(I:C) and IL‐13 (Figure 3A), suggesting that an alternative pathway was involved. Since IL‐13 also activated the IL‐13Rα2–PI3K–AKT pathway,^35^ and we noted that the PI3K inhibitor attenuated CCL5 production (Figure 3B), it is likely that the IL‐13Rα2‐PI3K‐AKT pathway is implicated in bringing about the synergistic effect observed. IRF3‐ and JAK1‐ knockdowns (Figure 3C,D) and treatment with the JAK1/2 inhibitor ruxolitinib (Figure 3E) also attenuated CCL5 production, further confirming that the TLR3–IRF3–IFNAR/JAK1–PI3K pathway is involved. Together, these data suggest that IL‐13 plus poly(I:C) synergistically induced the production of CCL5 in BEAS‐2B cells via the TLR3–IRF3–IFNR/JAK1–PI3K–AKT and IL‐13Rα2–PI3K pathways (Figure S3). However, this should also be examined for the IL‐4 signaling pathway.

### 3.4 Ruxolitinib is a potential therapeutic agent for severe eosinophilic asthma

**Figure 4:**
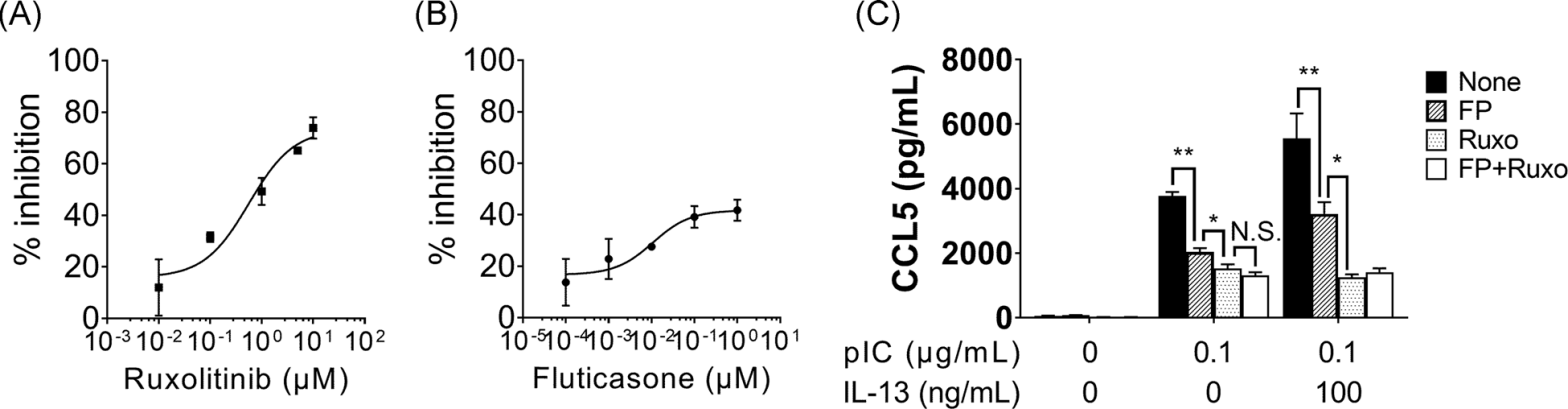
Ruxolitinib is a stronger inhibitor than fluticasone propionate for reducing CCL5 in BEAS‐2B cells treated with poly(I:C) and IL‐13. (A, B) BEAS‐2B cells were pre‐incubated with ruxolitinib (A) or fluticasone propionate (B) for 2 h, followed by stimulation with poly(I:C) (0.1 μg/ml). The maximal percentage (%) inhibition of ruxolitinib and fluticasone propionate against poly(I:C)‐induced CCL5 production was 73.4% and 41.7%, respectively. (C) BEAS‐2B cells were pre‐incubated with medium alone (control), fluticasone propionate (FP) (1 µM), ruxolitinib (Ruxo, 10 µM), or both, followed by stimulation with poly(I:C) with and without IL‐13. *p < .05, **p < .01, as compared to medium alone. We used one‐way ANOVA with post hoc Holm–Sidak's multiple tests to conduct selected pairwise comparisons of treatments. ANOVA, analysis of variance; CCL5, chemokine (C‐C motif) ligand 5; IL, interleukin; pIC, poly(I:C)

Based on our data, the IFNAR/JAK1–PI3K pathway was a key regulator of CCL5 production in our in vitro model of severe eosinophilic asthma with persistent type 2 inflammation. Ruxolitinib, a JAK1 inhibitor, is already clinically available. Hence, we assessed the therapeutic potential of ruxolitinib in comparison with FP, as measured by the ability of both drugs to inhibit CCL5 production after induction with both poly(I:C) and IL‐13. We first conducted a dose–response‐inhibition experiment, followed by a curve‐fitting analysis. These experiments estimated that the maximal inhibitory effects of ruxolitinib and FP against poly(I:C) induced‐CCL5 production were 73.4% and 41.7% (Figure 4A,B), respectively. We then conducted a head‐to‐head comparison between ruxolitinib (10 µM) and FP (1 µM). The selected concentrations of ruxolitinib were based on the maximal nontoxic concentrations as determined by preliminary cell‐toxicity experiments (Figure S4) and those of FP were based on concentrations given in previous reports.^36^ We found that ruxolitinib (10 µM) was a stronger inhibitor of CCL5 production than FP (1 µM) (Figure 4C). The ruxolitinib activity in this in vitro model supports the hypothesis that ruxolitinib is a better therapeutic option than FP for managing eosinophilic asthma with type 2 inflammation.

## 4 DISCUSSION

Here we have confirmed that poly(I:C) and IL‐13 synergistically enhance the production of CCL5 in bronchial epithelial cells. We regardthis experimental setup as a characteristic in vitro model of airway disease, because CCL5 production is clinically observed in patients with repeated viral infections, eosinophilic asthma, and persistent Th2‐type inflammation. The production of CCL5, induced by poly(I:C) plus IL‐13, was regulated by the TLR3–IRF3–IFNAR/JAK1–PI3K pathway; this may be due to activation of the IL‐13Rα2–PI3K pathways. This prompted our trial of ruxolitinib, a clinically available JAK1 inhibitor. We confirmed that ruxolitinib was a better inhibitor than FP for decreasing the synergistic production of CCL5 in vitro. Hence, ruxolitinib is a potential therapeutic agent for corticosteroid‐resistant severe eosinophilic asthma with a persistent type 2 inflammation phenotype. This is the first observation of the potential role of ruxolitinib in this setting.

Exacerbation of asthma is strongly associated with various RNA virus infections, including rhinovirus, RSV, and EV.^7^ Clinical and experimental data demonstrate that asthmatics have deficient immune responses to viruses and show higher viral loads and greater airway inflammation after viral infections than healthy subjects.^6^, ^37^, ^38^ The airway viral load in asthmatics correlates strongly with the severity of symptoms, hyperresponsiveness, and airflow limitations,^6^, ^39^ and is associated with airway eosinophilia. Furthermore, latent infection resulting from rhinovirus has been observed in 73% of stable asthmatics, in whom viral infection has been associated with eosinophilic lung infiltration and decreased lung function.^40^ In this study, we confirmed that poly(I:C), an RNA virus‐related TLR3 ligand, stimulated CCL5 production in bronchial epithelial cells. Taken together, it can be stated that patients with asthma are highly susceptible to viral infections, and both active and latent virus infection enhance airway eosinophilia and increase disease severity.

Viral infections cause the release of IL‐33 from bronchial epithelial cells, which enhances IL‐13 and IL‐5 production in naive (CD45RO^−^), activated (anti‐CD2/CD3/CD28‐stimulated), nonpolarized human CD1^+^T cells (Th0 cells) and ILC‐2s,^6^ providing a mechanism by which eosinophilic and Th2‐type asthma commonly overlap. Thus, IL‐33 is thought to promote type 2 inflammation.^6^ Similarly, it has been reported that IL‐37 and CC16are involved in the pathophysiology of bronchial asthma,^41^, ^42^ although we were able to in this study. However, we were able to demonstrate that the virus‐associated TLR3 ligand, along with either IL‐13 or IL‐4, enhances CCL5 production in bronchial epithelial cells. We consider this experimental setup to be an appropriate in vitro model of severe eosinophilic asthma associated with viral infection and persistent type 2 inflammation. In addition, since a previous study indicated that IL‐13 contributes more strongly to the pathogenesis of arising asthma than does IL‐4,^43^ we used IL‐13 instead of IL‐4 for the rest of the experiments. These findings demonstrate that viral infection in Th2‐type asthma results in a prominent increase in airway CCL5 production, which offers a potential mechanism through which severe eosinophilic asthma develops.

Multiple signal‐transduction pathways are involved in virus‐triggered CCL5 production in bronchial epithelial cells. dsRNA binds to TLR3 and activates downstream NF‐κB and IRF3 cascades, leading to CCL5 production.^44^ NF‐κB directly activates a variety of genes associated with inflammation,^45^ whereas IRF3 induces gene expression of IFNs, which in turn leads to the expression of ISGs via receptors for type I IFN.^46^ Our data demonstrate that IRF3‐knockdown strongly inhibited CCL5 production while Rel A‐knockdown and BAY11‐7082 failed to inhibit CCL5 production in BEAS‐2B cells. These results show that the TLR3 ligand stimulated the BEAS‐2B cells to produce CCL5 via IRF3 but not NF‐κB.

Øvrevik et al.^47^ have also reported that Rel A‐knockdown did not attenuate poly(I:C)‐induced CCL5 production in BEAS‐2B cells. However, other reports showed that poly(I:C) stimulated BEAS‐2B cells to produce CCL5 via both the NF‐κB and IRF3 pathways.^48^, ^49^ The reason for the discrepancy between our data and the existing literature remains unclear. There are two possible explanations for these differences^1^: we and Øvrevik et al.'s group used serum‐free medium for culturing BEAS‐2B cells, whereas others have used medium containing fetal bovine serum^47^, ^48^, ^49^; and^2^ the quality or purity of the poly(I:C) used may have differed between studies. The former is the more likely explanation, because the presence of fetal bovine serum leads to NF‐κB activation.^50^ The latter is less likely, since poly(I:C) is a synthesized molecule. Nevertheless, our data agreed with previously demonstrated findings that show that the TLR3‐IRF3 axis plays an important role in poly(I:C)‐induced CCL5 production in bronchial epithelial cells.

TLR3‐induced CCL5 production is mediated by type I IFNs^51^ and although we investigated whether alpha or beta IFNs were more important in this context, we were unable to answer this question (data not shown). IRF3 drives the expression of the gene encoding IFN and subsets of ISGs.^52^ Our results have demonstrated that poly(I:C)‐induced CCL5 production is attenuated by the neutralizing antibody against type I IFNs. This indicates that type I IFNs are involved in poly(I:C)‐induced CCL5 production, as reported previously.^53^ Type I IFNs bind to IFNAR, which bears JAK1 and TYK2. In this study, inhibitors of STAT1, STAT3, or Erk‐1/2, all of which are involved in the canonical IFNAR/JAK–STAT pathway, did not attenuate poly(I:C)‐induced CCL5 production, although LY294002 (an inhibitor of PI3K) did. This suggests that an alternative pathway, the IFNAR/JAK1–PI3K pathway, is involved in poly(I:C)‐induced CCL5 production, although we have not confirmed the nature of the interaction between IFNs, JAK1, and PI3K.

IL‐13 and IL‐4 both have augmented poly(I:C)‐induced CCL5 production in BEAS‐2B cells. The canonical receptor for IL‐13 is a type II receptor complex that consists of IL‐4Rα and IL‐13Rα1 anchoring JAK1 and TYK2, respectively.^54^ The noncanonical IL‐13 receptor is IL‐13Rα2, which lacks JAKs and TYK2^54^ but activates the PI3K and Erk 1/2 pathways.^55^, ^56^ The receptor for IL‐4 is a type I receptor complex that includes IL‐4Rα and a common γ‐chain that bears JAK1 and JAK3,^54^ which also activates the PI3K pathway in JAK1‐dependent manner.^57^, ^58^ Previous reports suggest that IL‐4 activates the IL‐4Rα–PI3K–Akt signaling pathway through a type I receptor, and this process is well characterized in hematopoietic cells.^59^, ^60^, ^61^ Another study showed that PI3K binds to TLR3, which subsequently phosphorylates IRF3 an essential step for TLR3–IRF3‐mediated gene induction.^62^ Thus, we speculate that IL‐4 is synergistically involved in poly(I:C)‐induced CCL5 production through PI3K activation. However, our experiments did not fully explain how IL‐4 activates the IL‐4Rα–PI3K–Akt pathway. Further research may thus be needed to clarify this relationship.

The augmenting of CCL5 production by IL‐13 remained when CCL5 production was not completely suppressed. We therefore suspect that PI3K and JAK1 are also involved in the augmentation effect. Contrary to expected findings, IL‐13 and poly(I:C)‐induced CCL5 production was independent of STAT6 but was dependent on PI3K, indicating that the synergy of the two stimulating cytokines was possibly induced by the IL‐13Rα2–PI3K pathway. This is consistent with a previous report whereby PI3K was found to bind to TLR3, which subsequently phosphorylated IRF3: an essential step for TLR3‐IRF3‐mediated gene induction.^62^ This signaling cascade can explain our observation that IL‐13‐augmented poly(I:C)‐induced CCL5 production, while IL‐13 alone did not stimulate CCL5 production. Moreover, the IFNAR/JAK1–PI3K pathway is also involved downstream of the TLR3–IRF3 pathway. Therefore, the JAK1–PI3K pathway is a key regulator for synergistic CCL5 production, which is a potential therapeutic target for severe asthma. Despite recent advances in medication for bronchial asthma, approximately 10% of those diagnosed have uncontrolled symptoms.^3^ A common phenotype of severe asthma includes persistent type 2 inflammation, which is characterized by sputum eosinophilia, high doses of inhaled corticosteroids, severe airflow limitations, and airway hyperresponsiveness.^3^ We have shown here, using an in vitro model, that ruxolitinib is potentially beneficial for treating severe eosinophilic asthmatics who require high doses of inhaled corticosteroids. Previous studies have shown that JAK1 is involved in many signaling cascades of IFNs, growth factors, and cytokines.^63^ This suggests that ruxolitinib has a possible advantage, given its ability to inhibit multiple pathways, versus monoclonal antibody therapies that target only a single molecule. FP, which is used in inhaled corticosteroids, is used as a therapeutic agent at dosages of under 1 μM.^36^ We showed that 10 μM of ruxolitinib is a more effective inhibitor of CCL5 production than 1 μM of FP. This indicates that ruxolitinib inhalation therapy may have therapeutic potential, since it carries a lower risk of systemic toxicity than FP. Further research is required to investigate the potential of ruxolitinib inhalation therapy, using intratracheal administration in animal models, as in one previous in vivo experiment that investigated the use of ruxolitinib to treat neutrophilic asthma.^64^

In conclusion, by treating BEAS‐2B cells with poly(I:C) and IL‐13, we confirmed the suitability of this an in vitro model of severe eosinophilic asthma with persistent type 2 inflammation, as confirmed by the increased CCL5 production. The efficacy of the JAK1 inhibitor ruxolitinib in inhibiting CCL5 production in this in vitro model suggests that ruxolitinib has therapeutic potential in severe eosinophilic asthma, which requires high‐dose inhaled corticosteroids. Further evaluation in animal models and clinical studies are necessary to confirm the suitability of ruxolitinib in patients.

## CONFLICT OF INTERESTS

The authors declare that htere are no conflict of interests.

## AUTHOR CONTRIBUTIONS

Conceptualization: Mitsuru Sada, Masato Watanabe, and Hajime Takizawa. Writing—original draft preparation: Mitsuru Sada. Writing—review and editing: Masato Watanabe, Hirokazu Kimura, and Hajime Takizawa. Supervised the data collection: Toshiya Inui, Keitaro Nakamoto, and provided expertize in respiratory medicine and revised it carefully from a professional point of view: Aya Hirata, Masuo Nakamura, Kojiro Honda, Takeshi Saraya, Daisuke Kurai and Haruyuki Ishii. All authors have read and agreed to the published version of the manuscript.

## Supporting information

Supporting information.

Supporting information.

Supporting information.

Supporting information.

Supporting information.

## Data Availability

The data that support the findings of this study are available from the corresponding author, upon reasonable request.
