# Supplementary figures and images for: Ruxolitinib inhibits poly(I:C) and type 2 cytokines‐induced CCL5 production in bronchial epithelial cells: A potential therapeutic agent for severe eosinophilic asthma
Source: Immun Inflamm Dis. 2021 Feb 3;9(2):363–73. doi: 10.1002/iid3.397 (PMC8127547; doi:10.1002/iid3.397)

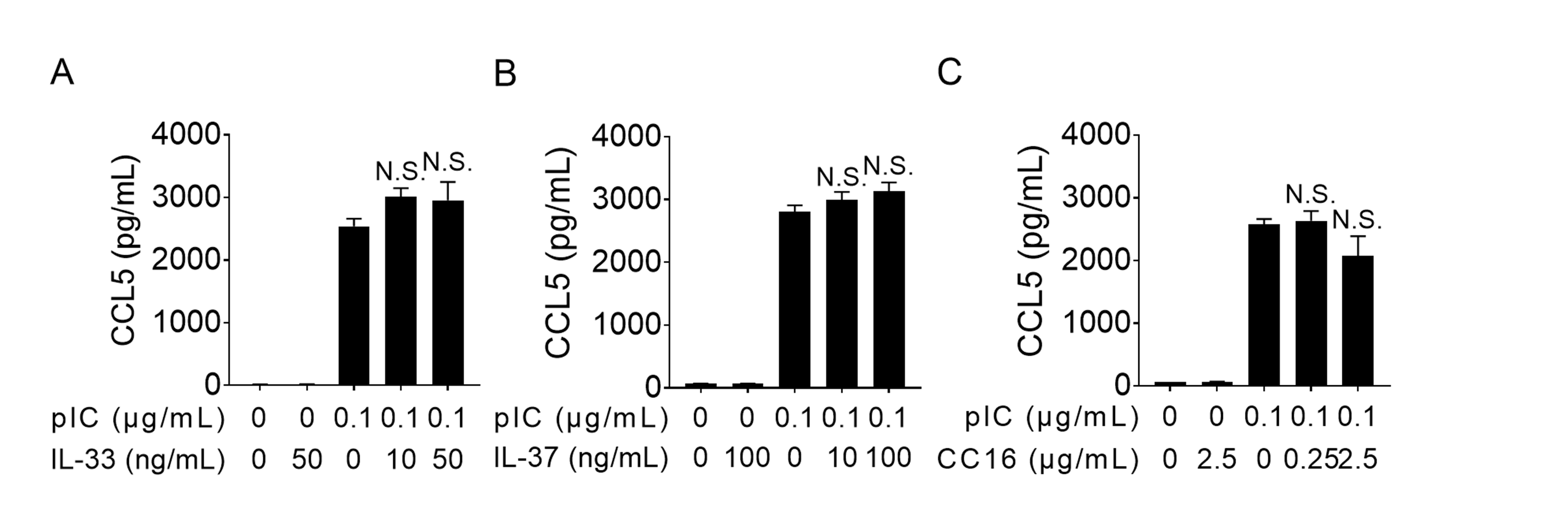

Supplement: Supplementary file 1 — Supporting information. [file IID3-9-363-s001.tif]

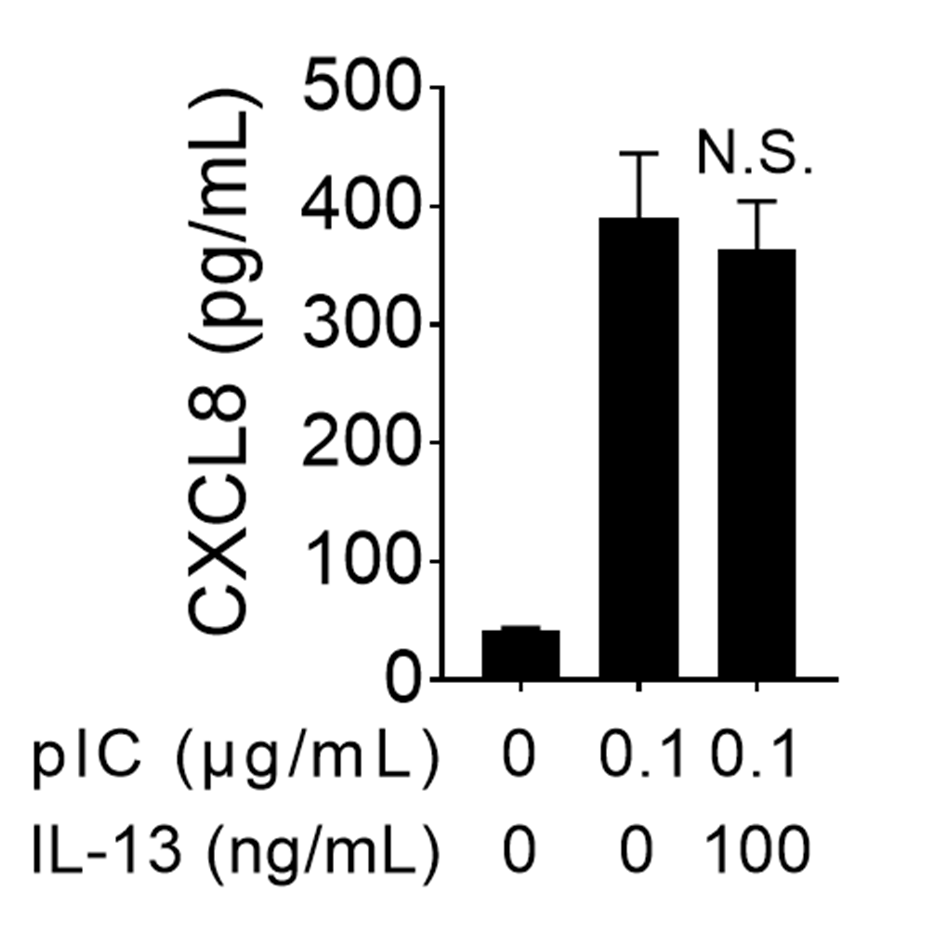

Supplement: Supplementary file 2 — Supporting information. [file IID3-9-363-s004.tif]

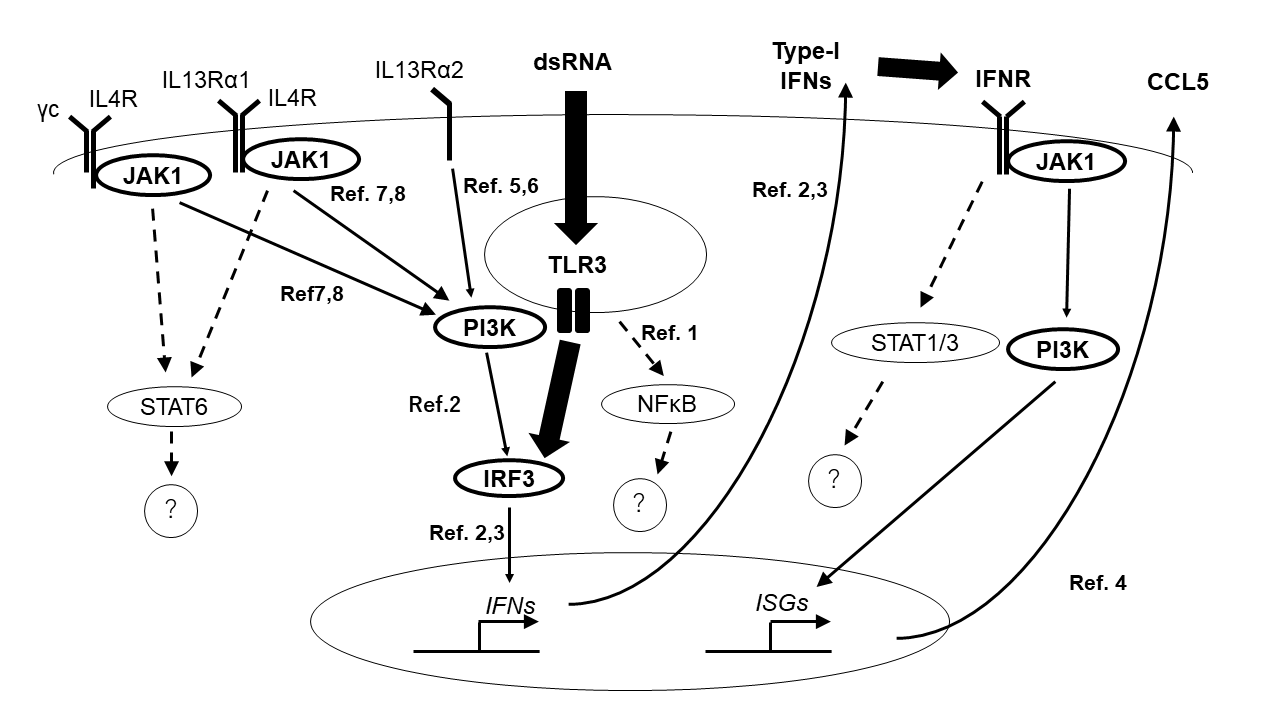

Supplement: Supplementary file 3 — Supporting information. [file IID3-9-363-s003.tif]

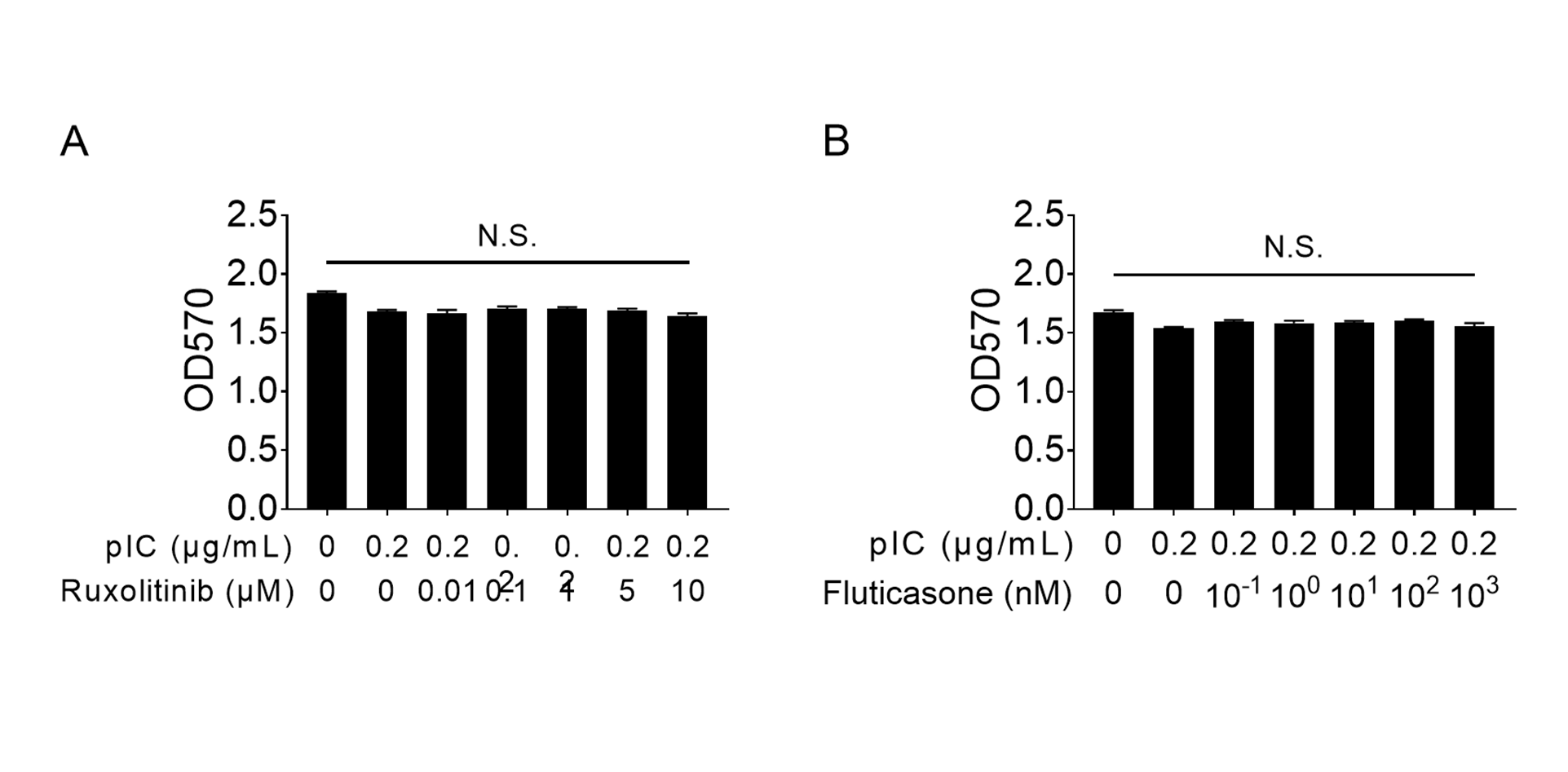

Supplement: Supplementary file 4 — Supporting information. [file IID3-9-363-s002.tif]
