# Supplementary material for: Ruxolitinib inhibits poly(I:C) and type 2 cytokines‐induced CCL5 production in bronchial epithelial cells: A potential therapeutic agent for severe eosinophilic asthma
Source: Immun Inflamm Dis. 2021 Feb 3;9(2):363–73. doi: 10.1002/iid3.397 (PMC8127547; doi:10.1002/iid3.397)
Supplement: Supplementary file 5 — Supporting information. [file IID3-9-363-s005.docx]

**Supplementary data**

**Supplementary Figure 1.** IL-33, IL-37, and CCL6 did not enhance poly (I:C)-induced CCL5 production in BEAS-2B cells.

BEAS-2B cells were stimulated with poly (I:C) and IL-33 (A), IL-37 (B), or CCL6 (C) for 24 h, after which the CCL5 concentration of the culture supernatant was evaluated.

*P*-values were calculated based on comparisons with poly (I:C) alone, using *post hoc* Holm-Sidak’s multiple tests to conduct selected pairwise comparisons.

pIC, poly (I:C). N.S., not significant.

**Supplementary Figure 2.** IL-13 did not enhance poly (I:C)-induced CXCL8 production in BEAS-2B cells.

BEAS-2B cells were stimulated with poly (I:C) and IL-13 for 24 h, after which the CXCL8 concentration of the culture supernatant was evaluated.

The *P*-value was calculated based on a comparison with poly (I:C) alone, using *post hoc* Holm-Sidak’s multiple tests to conduct selected pairwise comparisons.

pIC, poly (I:C). N.S., not significant.

**Supplementary Figure 3.** Schematic of the signal transduction mechanisms involved in poly (I:C)- and IL-13-induced CCL5 production in bronchial epithelial cells.

Thick solid arrows, cascades that our experiments have demonstrated. Thin solid arrows, cascades that our experiments have suggested. Dashed arrows, cascades that our experiments have not demonstrated. Refs 1–8, literature indicating what that cascade is involved in.

**References**

1. Melkamu, T., H. Kita, and S.M. O'Grady, *TLR3 activation evokes IL-6 secretion, autocrine regulation of Stat3 signaling and TLR2 expression in human bronchial epithelial cells.* Journal of Cell Communication and Signaling, 2013. **7**(2): p. 109-118.

2. Schneider, W.M., M.D. Chevillotte, and C.M. Rice, *Interferon-stimulated genes: a complex web of host defenses.* Annual Review of Immunology, 2014. **32**: p. 513-545.

3. Perng, Y.-C. and D.J. Lenschow, *ISG15 in antiviral immunity and beyond.* Nature Reviews Microbiology, 2018. **16**(7): p. 423-439.

4. Qaisar, N., et al., *A Critical Role for the Type I Interferon Receptor in Virus-Induced Autoimmune Diabetes in Rats.* Diabetes, 2017. **66**(1): p. 145-157.

5. Mannon, P. and W. Reinisch, *Interleukin 13 and its role in gut defence and inflammation.* Gut, 2012. **61**(12): p. 1765-1773.

6. Bartolomé, R.A., et al., *IL13 Receptor α2 Signaling Requires a Scaffold Protein, FAM120A, to Activate the FAK and PI3K Pathways in Colon Cancer Metastasis.* Cancer Research, 2015. **75**(12): p. 2434-2444.

7. Burfoot, M.S., et al., *Janus kinase-dependent activation of insulin receptor substrate 1 in response to interleukin-4, oncostatin M, and the interferons.* Journal of Biological Chemistry, 1997. **272**(39): p. 24183-24190.

8. Gadani, S.P., et al., *IL-4 in the brain: a cytokine to remember.* The journal of immunology, 2012.　　　**189**(9): p. 4213-4219.

**Supplementary Figure 4.** Ruxolitinib and fluticasone propionate (FP) did not exhibit a cytotoxic effect at concentrations within the measuring range in BEAS-2B cells.

BEAS-2B cells were stimulated with poly (I:C) and ruxolitinib (A) or FP (B) for 24 h, after which their cytotoxic effect was evaluated via a tetrazolium salt assay.

*P*-values were calculated based on comparisons with poly (I:C) alone, using *post hoc* Holm-Sidak’s multiple tests to conduct selected pairwise comparisons.

pIC, poly (I:C). OD570, optical density at 570 nm.
